# Supplementary figures and images for: NFAT signaling is indispensable for persistent memory responses of MCMV-specific CD8+ T cells
Source: PLoS Pathog. 2024 Feb 12;20(2):e1012025. doi: 10.1371/journal.ppat.1012025 (PMC10890734; doi:10.1371/journal.ppat.1012025)

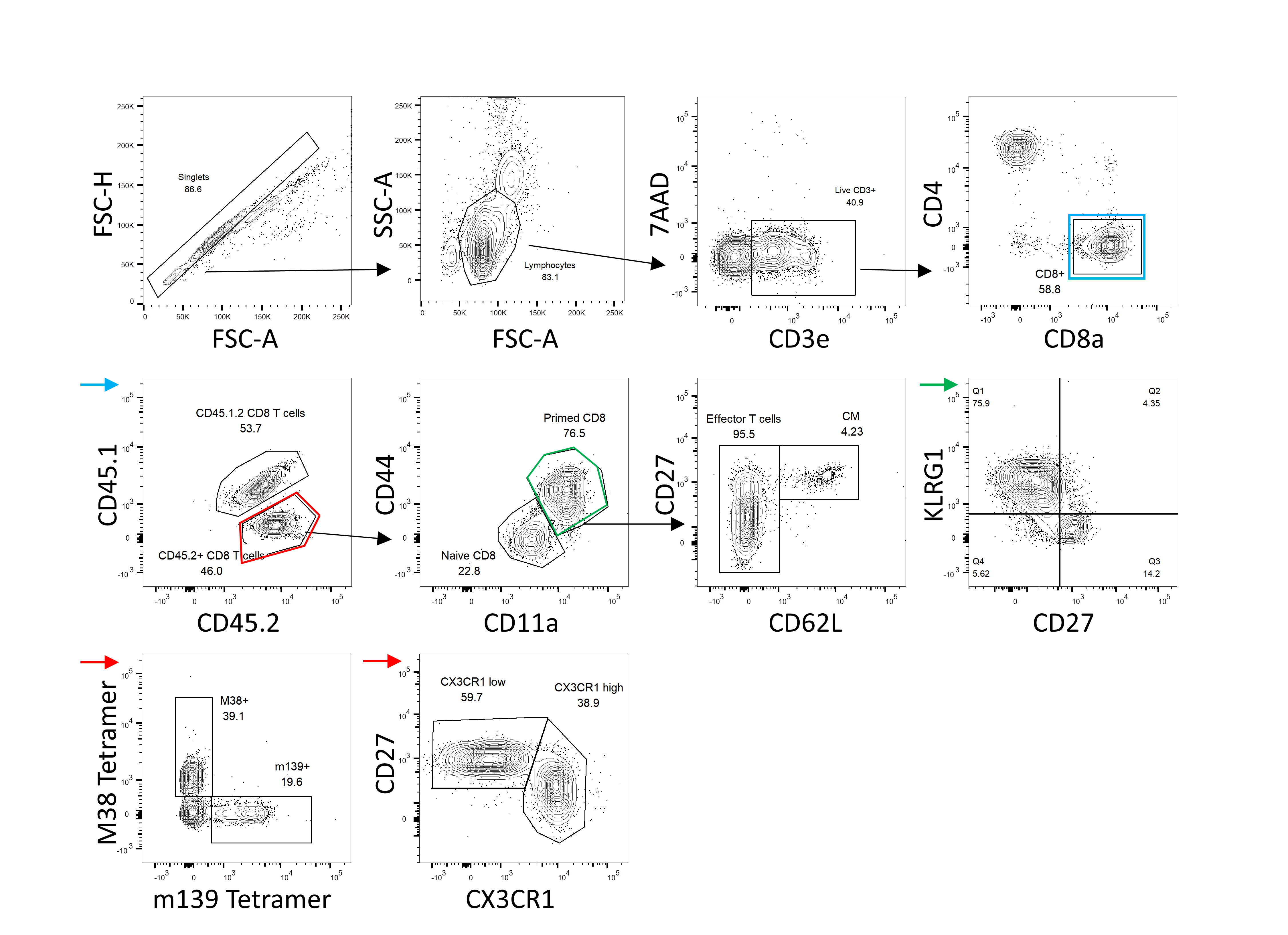

Supplement: S1 Fig — Following singlet gating, lymphocytes were selected with forward/side scatter parameters. Live CD3+ cells were identified by excluding 7AAD stained cells, and CD8+ T cells were selected by CD8a expression. Next, CD8+ T cells (blue gate and arrow) were gated into CD45.2+ single and CD45.1+CD45.2+ double positive populations in mixed bone chimeric animals. Population arising from each bone marrow was progressively gated to define naive and primed CD8+ T cells based on CD44 and CD11a expression. Central memory cells were distinguished from effector cells by CD62L and CD27 expression within primed population. Similarly, short lived effector and memory cell populations (SLEC and Mem) were gated according to KLRG1 and CD27 expression within primed CD8+ T cells (green gate and arrow). Tetramer+ (M38+ and m139+) and CX3CR1+ populations were defined by gating directly on CD45.2+ CD8+ T cells (red gate and arrow) or control CD45.1+CD45.2+ CD8+ T cell populations. (TIF) [file ppat.1012025.s001.tif]

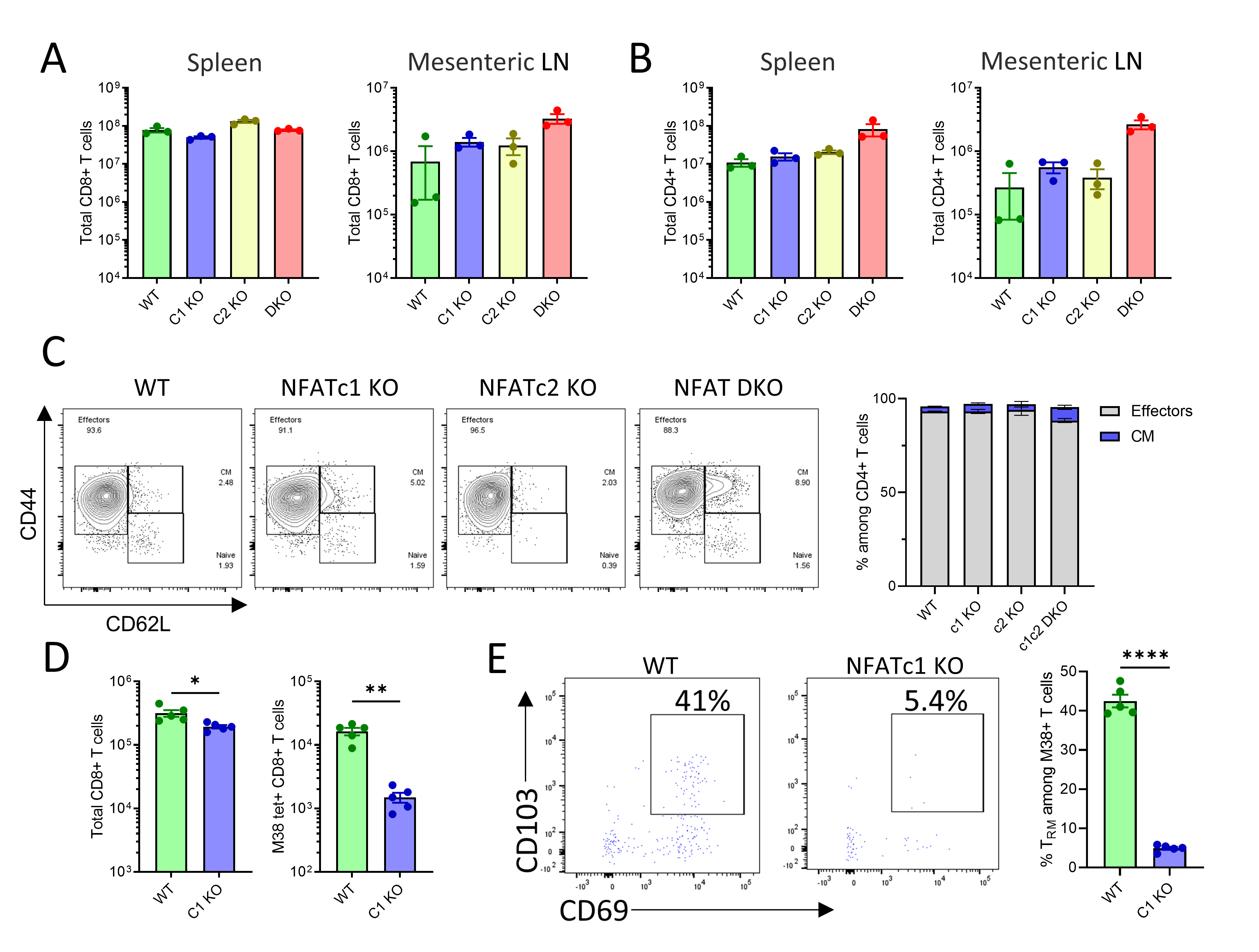

Supplement: S2 Fig — (A-C) Animals lacking either NFATc1, NFATc2 or both were infected with 106 PFU of MCMV intraperitoneally and T cells responses analyzed at 7 dpi. CD8+ T cell (A) and CD4+ T cell (B) responses in spleen and mLN. (C) Flow-cytometric plots show effector, central memory (CM) and naïve CD4+ T cells populations (left). Kinetics of CD4+ T cell effector and CM populations in spleen are shown on right, error bars are SEM (n = 3). (D-E) WT and NFATc1 KO animals were infected with 106 PFU of MCMV via intranasal (i.n.) route and CD8+ T cell responses in lungs were analyzed at 30 dpi. (D) Total CD8+ T cell and M38 tetramer+ responses in lungs following i.n. virus infection. (E) Resident memory T cells (CD69+CD103+) among virus specific (M38 tetramer+) CD8+ T cells is shown. Statistically significant differences are highlighted; *, p < 0.05; **, p < 0.01; ****, p < 0.0001; (Mann-Whitney U Test). (TIF) [file ppat.1012025.s002.tif]

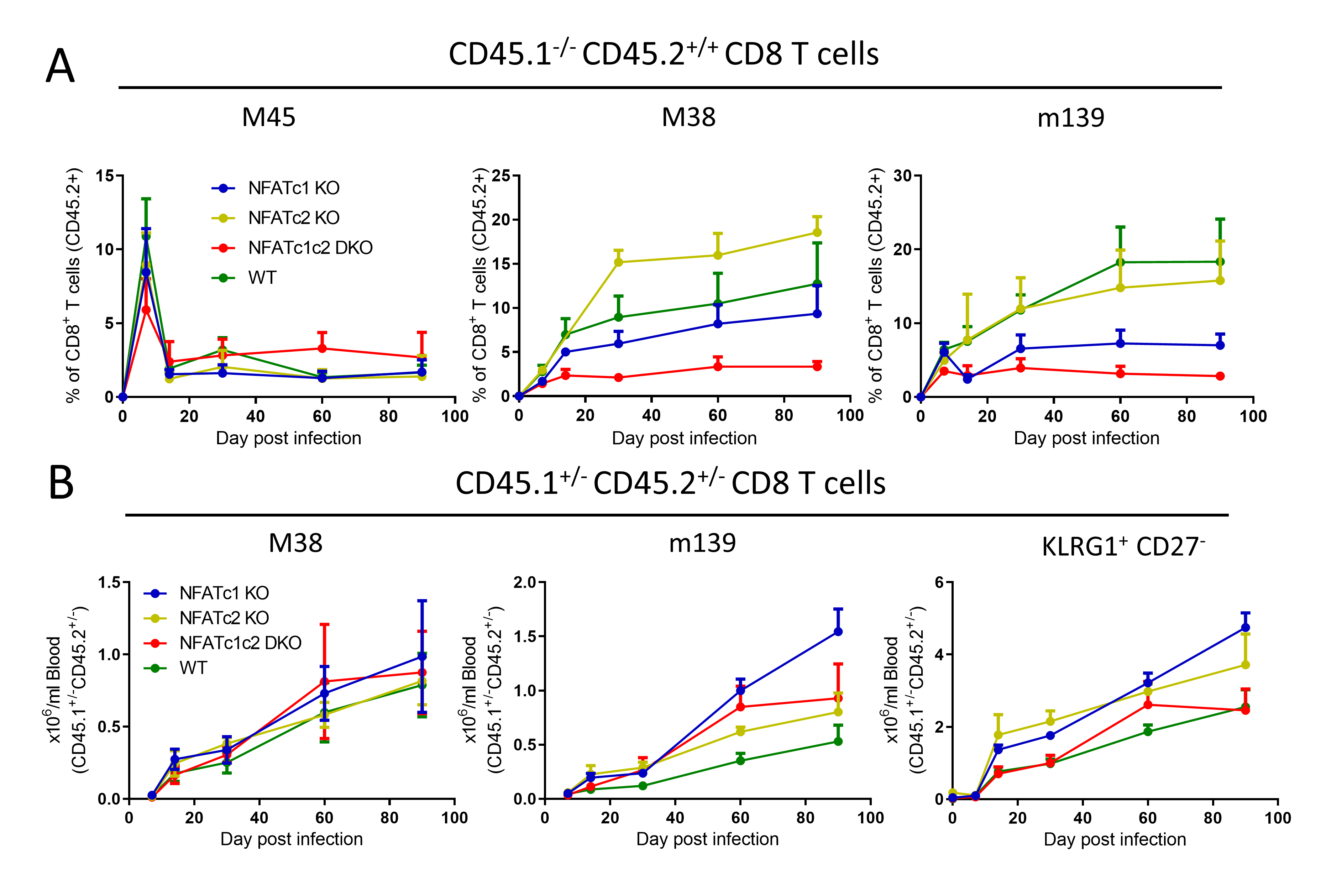

Supplement: S3 Fig — Lethally irradiated mice were reconstituted with BM (1:1) of NFATc1 KO, NFATc2 KO, NFATc1c2 DKO or wildtype BM (CD45.2+/+) with control wildtype BM (CD45.1+/-CD45.2+/-). CD8+ T cell response kinetics were monitored for 90 days following intraperitoneal MCMV infection with 106 PFU. (A) Relative frequency of tetramer+ cells among CD45.2+/+ CD8+ T cells. (B) Absolute size of SLEC (KLRG1+ CD27-) and tetramer specific responses from control CD45.1+/-CD45.2+/- population. Data are pooled from two experiments and for each group n≥6. Statistically significant differences are highlighted; *, p < 0.05; **, p < 0.01; ***, p < 0.001; (Mann-Whitney U Test); mean ± SEM values are plotted. (TIF) [file ppat.1012025.s003.tif]

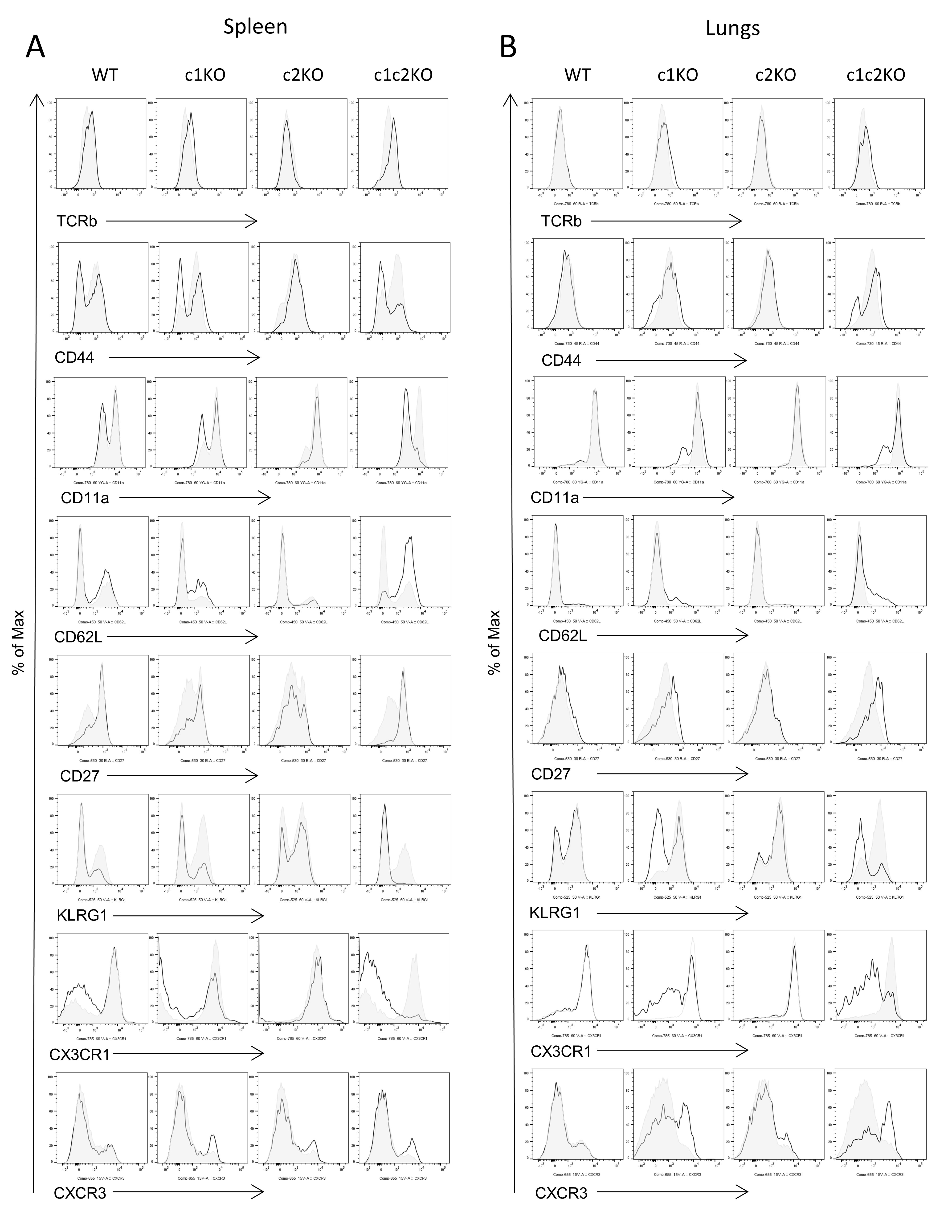

Supplement: S4 Fig — Lymphocytes from spleen (A) or lungs (B) of mice infected for 3 months were stained for CD45.1, CD45.2 and CD8 expression as well as the indicated cell-surface molecules. The plots shown are gated on CD45.1+/-CD45.2+/- CD8+ T cells (shaded histogram) or CD45.1-/-CD45.2+/+ CD8+ T cells (black line) from the same sample. Data are representative of at least six individual mice per stain and two independent experiments. (TIF) [file ppat.1012025.s004.tif]

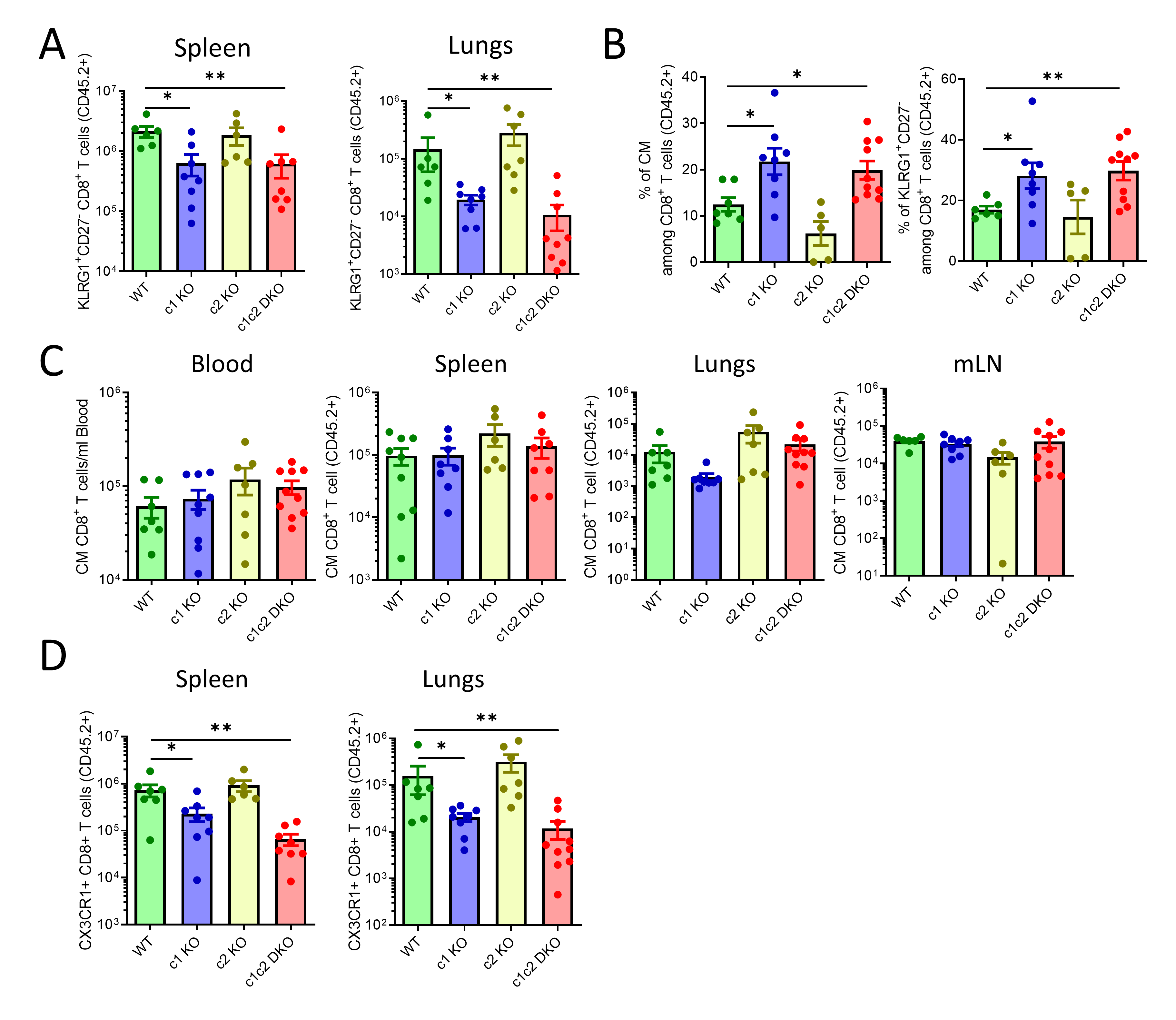

Supplement: S5 Fig — Mixed bone marrow chimeric animals were infected with 106 PFU of MCMV and sacrificed at 90 dpi. (A) Absolute count of KLRG1+CD27- CD8+ T cells in spleen and lungs of BMC animals at 90 dpi. (B) Frequency of CM and KLRG1-CD27+ T cells among primed CD45.2+/+ CD8+ T cells (CD44+CD11a+) from mesenteric LN of chronically infected mice. (C) Quantification of CM CD8+ T cells from CD45.2+/+ compartment in blood, spleen, lungs and mesenteric LN. (D) Absolute count of CX3CR1+ CD8+ T cells in spleen and lungs of BMC animals at 90 dpi. Data are pooled from two independent experiments and each dot represent one mouse. Statistically significant differences are highlighted; *, p < 0.05; **, p < 0.01; ***, p < 0.001; (Mann-Whitney U Test); mean ± SEM values are plotted. (TIF) [file ppat.1012025.s005.tif]

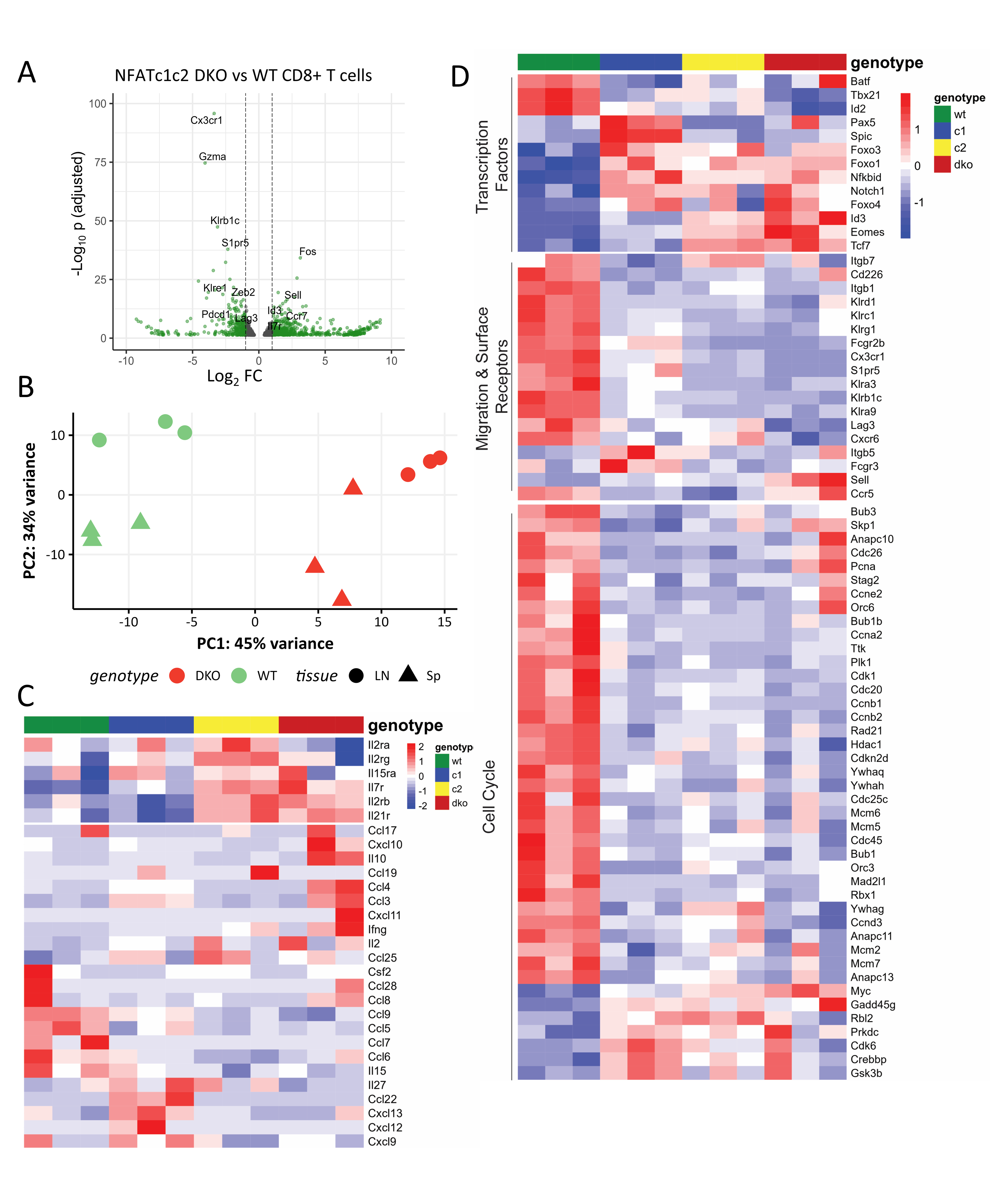

Supplement: S6 Fig — Naïve OTI T cells (104) were transferred to congenic animals and activated by acute MCMV infection. Animals were sacrificed at 7 dpi and transcriptional analysis was performed. (A) Volcano plot shows genes that are differentially regulated in NFATc1c2 DKO OTI cells as compared to WT cells. (B) Principal component analysis of RNA sequencing samples from WT and NFATc1c2 DKO CD8+ T cells from spleen and lymph nodes (LN). Replicates of the same genotype and tissue are indicated by similar color and shape, respectively. (C) Heatmap shows expression of selected cytokine and cytokine receptor genes in OTI cells that lack NFATc1, NFATc2 or both. (D) Heatmap shows expression of selected transcription factors, surface receptors and cell cycle regulation genes from CD8+ T cells. Color shows Z-score differences. (TIF) [file ppat.1012025.s006.tif]

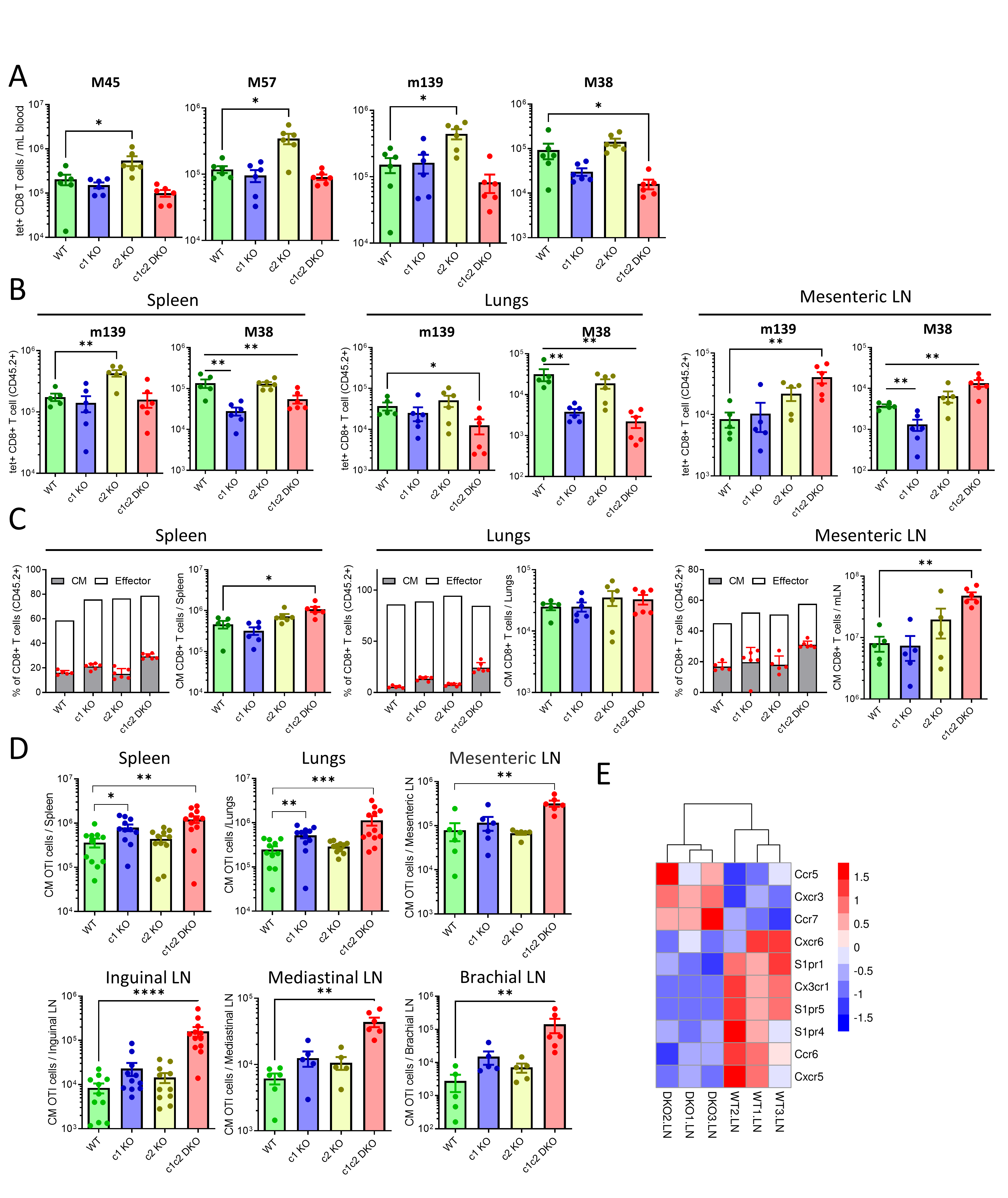

Supplement: S7 Fig — (A-C) Mixed bone marrow chimeric animals were infected with 106 PFU of MCMV and sacrificed at 7 dpi. Absolute count of different tetramer specific CD45.2+/+ CD8+ T cells in blood (A), spleen, lungs and mesenteric LN (B). (C) Relative and absolute count of CM CD45.2+/+ CD8+ T cells in spleen, lungs and mesenteric LN. (D-F) 104 naïve OTI T cells were transferred to congenic animals and activated by MCMV infection. (D) Absolute count of CM OTI T cells in different organs at 7 dpi is shown. (E) Selected genes involved in T cell migration are shown in heatmap. Data are pooled from at least two experiments and each dot represents one mouse; mean ± SEM values are plotted. (TIF) [file ppat.1012025.s007.tif]
